# Supplementary material for: The Diverse Mycorrizal Morphology of Rhododendron dauricum, the Fungal Communities Structure and Dynamics from the Mycorrhizosphere
Source: J Fungi (Basel). 2024 Jan 14;10(1):65. doi: 10.3390/jof10010065 (PMC10817234; doi:10.3390/jof10010065)
Supplement: Supplementary file 1 [file jof-10-00065-s001.zip › Figure S1 Reference diagram for mycorrhizal type identification.pdf]

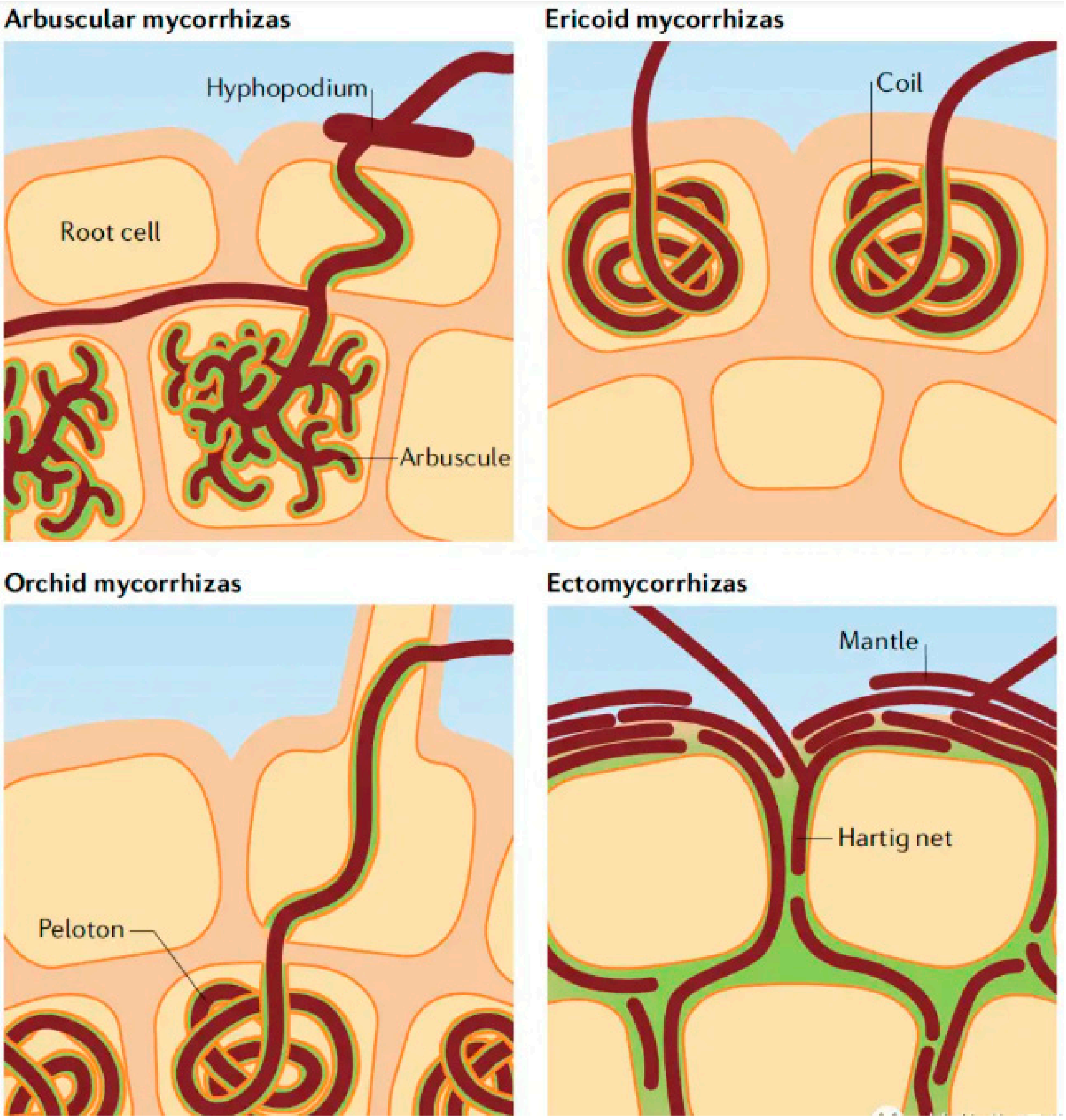

Figure S1. Reference diagram for mycorrhizal type identification

Note: Reference (Genre.; Andrea.; Lanfranco.; Luisa.; Perotto.; Silvia. Unique and common traits in mycorrhizal symbioses. Nature Reviews Microbiology. 2020, 18, 649-660.)
