# Supplementary material for: The Diverse Mycorrizal Morphology of Rhododendron dauricum, the Fungal Communities Structure and Dynamics from the Mycorrhizosphere
Source: J Fungi (Basel). 2024 Jan 14;10(1):65. doi: 10.3390/jof10010065 (PMC10817234; doi:10.3390/jof10010065)
Supplement: Supplementary file 1 [file jof-10-00065-s001.zip › Table S1 Dominant fungal genera.pdf]

Table S1. The dominant fungal genera and their relative abundance of eight samples		
**Samples**	**Genus**	**The relative abundance**
SAQT	*Russula*	31.23%
	Cortinarius	16.49%
	Umbelopsis	3.59%
	Mortierella	2.04%
	Tricholoma	1.33%
SAHT	*Tricholoma*	42.35%
	Umbelopsis	23.38%
	Russula	18.05%
	Mortierella	2.88%
	Trichoderma	2.49%
SAQG	*Russula*	22.46%
	Cortinarius	15.81%
	Phialocephala	5.48%
	Tricholoma	3.22%
	Cladophialophora	1.29%
SAHG	*Russula*	31.92%
	Tricholoma	6.24%
	Mortierella	5.05%
	Trichoderma	4.79%
	Cladophialophora	4.25%
	Cortinarius	2.47%
	Umbelopsis	1.94%
SBQT	*Russula*	24.61%
	Mortierella	9.36%
	Umbelopsis	6.37%
	Tricholoma	3.64%
	Cladophialophora	1.81%
SBHT	*Russula*	40.08%
	Hydnellum	37.43%
	Umbelopsis	2.15%
	Cortinarius	1.23%
SBQG	*Phialocephala*	26.05%
	Lachnum	8.75%
	Cladophialophora	6.94%
	Mortierella	6.14%
	Trichoderma	2.85%
	Russula	1.58%
	Umbelopsis	1.19%
SBHG	*Phialocephala*	22.71%
	Trichoderma	5.76%
	Cladophialophora	5.51%
	Lachnum	4.67%
	Hydnellum	4.50%
	Mortierella	2.92%

Note: SAQT represents soil samples from SA site in June to July, SAHT represents soil samples from SA site in August to September, SAQG represents mycorrhizal samples from SA site in June to July, SAHG represents mycorrhizal samples from SA site in August to September; SBQT represents soil samples from SB site in June to July,

SBHT represents soil samples from SB site in August to Sep-tember, SBQG represents mycorrhizal samples from SB site in June to July, SBHG represents my-corrhizal samples from SB site in August to September.
