# Supplementary material for: The Diverse Mycorrizal Morphology of Rhododendron dauricum, the Fungal Communities Structure and Dynamics from the Mycorrhizosphere
Source: J Fungi (Basel). 2024 Jan 14;10(1):65. doi: 10.3390/jof10010065 (PMC10817234; doi:10.3390/jof10010065)
Supplement: Supplementary file 1 [file jof-10-00065-s001.zip › Table S2 Betweenness centrality of each point in the network diagram.pdf]

Table S2. Betweenness centrality of each point in the network diagram

Node_id	Betweenness centrality
Paratritirachium_S	568
Delastria_S	556
Serendipita_S	510
Amanita_S	360
Hydnellum_R	306
Phellodon_S	243
Peziza_S	228
Gymnopus_S	228
Piloderma_S	183
Pseudotomentella_R	177
Mortierella_S	150
Cladophialophora_S	147
Chaetomium_S	114
Leohumicola_S	108
Vanrija_S	92
Penicillium_S	83
Laccaria_S	82
Umbelopsis_R	82
Exophiala_S	81
Scleroderma_S	78
Suillus_S	78
Articulospora_S	78
Inocybe_S	77
Russula_S	73
Absidia_S	72
Rhinocladiella_S	72
Gibberella_S	71
Leptodontidium_S	70
Cortinarius_R	70
Geomyces_S	66
Mycosphaerella_S	57
Hydnellum_S	56
Ilyonectria_S	56
Talaromyces_S	55
Pseudotomentella_S	53
Hygrophorus_S	51
Bifiguratus_S	43
Humicola_S	42
Umbelopsis_S	36
Archaeorhizomyces_S	36
Trichocladium_S	34
Pseudogymnoascus_S	33
Oidiodendron_S	32
Tomentella_S	31
Trichoderma_S	28
Geminibasidium_S	22
Tausonia_S	22
Solicoccozyma_S	21

Paraboeremia_S	21
Paraphoma_S	18
Tricholoma_R	18
Cenococcum_S	12
Tricholoma_S	10
Fusarium_S	9
Cryptococcus_S	9
Aspergillus_S	8
Cutaneotrichosporon_S	8
Mycena_S	7
Elaphomyces_S	6
Russula_R	6
Pichia_S	5
Sistotrema_S	2
Thelephora_S	2
Mycena_R	2
Lactifluus_S	1
Marasmius_S	1
Lachnum_R	1
Sebacina_S	0
Saitozyma_S	0
Cortinarius_S	0
Amphinema_S	0
Tomentellopsis_S	0
Lactarius_S	0
Phialocephala_S	0
Devriesia_S	0
Clavulina_S	0
Knufia_S	0
Mucor_S	0
Apiotrichum_S	0
Coniochaeta_S	0
Trechispora_S	0
Boletus_S	0
Holtermanniella_S	0
Sagenomella_S	0
Tuber_S	0
Gyroporus_S	0
Lachnum_S	0
Astraeus_S	0
Membranomyces_S	0
Xylogone_S	0
Leotia_S	0
Verticillium_S	0
Thelonectria_S	0
Clavulinopsis_S	0
Rhizopogon_S	0
Dasyspora_S	0
Cephalotrichum_S	0
Pochonia_S	0

Phialocephala_R	0
Tomentella_R	0
Oidiodendron_R	0
Cenococcum_R	0

Note: Genus_R represents genus from mycorrhizal samples, Genus_S represents genus from soil samples.
